# European Sea Bass (*Dicentrarchus labrax*) Immune Status and Disease Resistance Are Impaired by Arginine Dietary Supplementation

**DOI:** 10.1371/journal.pone.0139967

**Published:** 2015-10-08

**Authors:** Rita Azeredo, Jaume Pérez-Sánchez, Ariadna Sitjà-Bobadilla, Belén Fouz, Lluis Tort, Cláudia Aragão, Aires Oliva-Teles, Benjamín Costas

**Affiliations:** 1 Centro Interdisciplinar de Investigação Marinha e Ambiental (CIIMAR), Universidade do Porto, Rua dos Bragas 289, 4050-123, Porto, Portugal; 2 Departamento de Biologia, Faculdade de Ciências da Universidade do Porto (FCUP), 4169-007, Porto, Portugal; 3 Departament de Biologia Cel·lular, Fisiologia Animal i Immunologia, Universitat Autònoma de Barcelona, Bellaterra, Spain; 4 Nutrigenomics and Fish Growth Endocrinology Group, Institute of Aquaculture Torre de la Sal, IATS-CSIC, 12595 Ribera de Cabanes, Castellón, Spain; 5 Fish Pathology Group, Institute of Aquaculture Torre de la Sal, IATS-CSIC, 12595 Ribera de Cabanes, Castellón, Spain; 6 Department of Microbiology and Ecology, Faculty of Biology, University of Valencia, Dr Moliner 50, 46100 Burjassot, Valencia, Spain; 7 Centro de Ciências do Mar, Universidade do Algarve, Campus de Gambelas, edf. 7, 8005-139, Faro, Portugal; The University of Plymouth, UNITED KINGDOM

## Abstract

Infectious diseases and fish feeds management are probably the major expenses in the aquaculture business. Hence, it is a priority to define sustainable strategies which simultaneously avoid therapeutic procedures and reinforce fish immunity. Currently, one preferred approach is the use of immunostimulants which can be supplemented to the fish diets. Arginine is a versatile amino acid with important mechanisms closely related to the immune response. Aiming at finding out how arginine affects the innate immune status or improve disease resistance of European seabass (*Dicentrarchus labrax*) against vibriosis, fish were fed two arginine-supplemented diets (1% and 2% arginine supplementation). A third diet meeting arginine requirement level for seabass served as control diet. Following 15 or 29 days of feeding, fish were sampled for blood, spleen and gut to assess cell-mediated immune parameters and immune-related gene expression. At the same time, fish from each dietary group were challenged against *Vibrio anguillarum* and survival was monitored. Cell-mediated immune parameters such as the extracellular superoxide and nitric oxide decreased in fish fed arginine-supplemented diets. Interleukins and immune-cell marker transcripts were down-regulated by the highest supplementation level. Disease resistance data were in accordance with a generally depressed immune status, with increased susceptibility to vibriosis in fish fed arginine supplemented diets. Altogether, these results suggest a general inhibitory effect of arginine on the immune defences and disease resistance of European seabass. Still, further research will certainly clarify arginine immunomodulation pathways thereby allowing the validation of its potential as a prophylactic strategy.

## Introduction

The biggest challenges regarding European sea bass (*Dicentrarchus labrax*) Mediterranean aquaculture are the severe bacterial outbreaks occurring throughout the year [1]. Among them, vibriosis causes the highest mortality rates and, consequently, great economic losses. *Vibrio anguillarum* is the main aetiological agent of vibriosis often leading to a septicaemia as well as haemorrhages and exophthalmia [2]. Immunization by vaccination has been applied [3], but distinct and complementary approaches are recommended in order to successfully and cost-efficiently produce European sea bass. Indeed, formulation of fish feeds must address other issues concerning fish welfare and health besides promoting optimum growth. Supplementing fish diets with key ingredients is a strategy often used in aquaculture to improve a selected trait. Such diets (functional diets) can also be used to improve fish defences and thereby, avoid high mortalities during a viral or bacterial incursion episode. Vitamins, prebiotics, probiotics and pigments such as xanthophylls and astaxanthins have been utilized as supplements for these diets [4–6]. More recently, amino acids (AA) have been employed in studies of immunomodulation [7–9], but such knowledge is still scarce.

Arginine is one of the most versatile AA, hence it represents a good candidate for inclusion in functional diets. Arginine requirements among different fish species are generally high given its great contribution to proteins composition and body fluids and also the almost total absence of its *de novo* synthesis, as this is an essential AA [10]. Similarly to higher vertebrates, fish can produce nitric oxide (NO) and ornithine from arginine using the enzymes inducible nitric oxide synthase (iNOS) and arginase, respectively [11]. When produced by phagocytes, NO is used against pathogens, acting as an oxidant that compromises structures integrity and function. Such actions represent self-damage as well, and when combined with superoxide anion, the toxicity dangerously increases [12]. Furthermore, arginine can enhance cell proliferation by fuelling polyamine biosynthesis through provision of ornithine [13]. Altogether, upon infection, these metabolic pathways may stimulate the inflammatory response. However, arginine can also mediate immunosuppressive mechanisms. In mammals, T-cell activation and function is dictated by arginine metabolism in myeloid suppressor cells (MSC) [11]. These cells metabolize arginine with either arginase or iNOS according to different stimuli and the derived products are directly involved in the suppression of T-cell functions. When arginine was added both to a primary enterocyte culture media or to the diet of Jian carp (*Cyprinus carpio* var. Jian), an inhibition of the LPS-induced inflammatory response was detected [14].

While being aware of the high importance of arginine as an essential constituent for growth, it would be of major interest to fully determine the mechanisms of arginine immunomodulation. Therefore, this study aims to decipher to what extent dietary arginine supplementation may modulate the European sea bass immune response and disease resistance against *Vibrio anguillarum*.

## Material and Methods

### 2.1 Diet formulation

Three plant protein-based diets with a reduced inclusion level of fish meal and fish soluble protein concentrates (12.2%) were formulated and manufactured by Sparos Lda. (Olhão, Portugal). A blend of fish oil (6.2%): rapeseed oil (4.2%): palm oil (4.2%) was used as dietary lipid source. The control diet (CTRL) was formulated to include an indispensable AA concentration meeting the ideal pattern estimated for European sea bass [15]. The two other diets were identical to the CTRL but supplemented with L-arginine at 1% or 2% (Arg1 and Arg2, respectively) at the expenses of wheat gluten. Main ingredients were ground (below 250 μm) in a micropulverizer hammer mill (SH1; Hosokawa Micron, B.V., Doetinchem, The Netherlands). Powder ingredients and oils were then mixed according to the target formulation in a paddle mixer (RM90; Mainca, S.L., Granollers, Spain). All diets were manufactured by temperature-controlled extrusion (pellet sizes: 1.5 and 2 mm) by means of a low-shear extruder (P55; Italplast, S.r.l., Parma, Italy). Upon extrusion, all feed batches were dried in a convection oven (OP 750-UF; LTE Scientifics, Oldham, UK) for 4h at 45°C. Formulation and proximate composition of experimental diets are presented in Table 1.

**Table 1:** Ingredients and proximal composition of experimental diets.

	Experimental diets		
	CTRL	Arg1	Arg2
*Ingredients (% dry matter)*			
Fishmeal Super Prime^1^	10.0	10.0	10.0
Fish soluble protein concentrate 90^2^	2.0	2.0	2.0
Fish gelatin^3^	0.2	0.2	0.2
Soy protein concentrate^4^	20.0	20.0	20.0
Pea protein concentrate^5^	7.0	7.0	7.0
Wheat gluten^6^	9.6	8.6	7.6
Corn gluten^7^	13.0	13.0	13.0
Soybean meal^8^	6.0	6.0	6.0
Rapeseed meal^9^	5.0	5.0	5.0
Wheat meal	7.0	7.0	7.0
Fish oil^10^	6.2	6.2	6.2
Rapeseed oil^11^	4.2	4.2	4.2
Palm oil^12^	4.2	4.2	4.2
Vitamin and mineral premix^13^	1.0	1.0	1.0
Choline chloride	0.2	0.2	0.2
Soy lecithin	1.6	1.6	1.6
Antioxidant^14^	0.2	0.2	0.2
Mono calcium phosphate^15^	2.1	2.1	2.1
L-Arginine		1.0	2.0
L-Lysine	0.4	0.4	0.4
DL-Methionine	0.1	0.1	0.1
*Proximate analyses (% dry weight)*	**CTRL**		
Dry matter	91.0	91.9	91.7
Crude protein	51.5	54.2	55.9
Crude fat	22.3	21.5	21.7
Ash	7.0	7.2	7.1
Gross Energy (kJ g^-1^ DM)	26.3	27	26.6

^1^ Peruvian fishmeal: 71% crude protein (CP), 11% crude fat (CF), EXALMAR, Peru

^2^ CPSP 90 fish soluble protein concentrate: 84% CP, 12% CF, Sopropêche, France.

^3^ Pharma Grade bloom 240: 92% CP, LAPI Gelatine SPA, Italy

^4^ Soycomil P: 65% CP, 0.8% CF, ADM, The Netherlands.

^5^ Lysamine GP: 78% CP, 8% CF, ROQUETTE, France.

^6^ VITAL: 85.7% CP, 1.3% CF, ROQUETTE, France.

^7^ Corn gluten feed: 61% CP, 6% CF, COPAM, Portugal.

^8^ Solvent extracted dehulled soybean meal: 47% CP, 2.6% CF, SORGAL SA, Portugal.

^9^ Defatted rapeseed meal: 36% CP, 2% CF, SORGAL SA, Portugal.

^10^ COPPENS International, The Netherlands.

^11^ Henry Lamotte Oils GmbH, Germany.

^12^ Crude palm oil: Gustav Heess GmbH, Germany.

^13^ Premix for marine fish, PREMIX Lda, Portugal. Vitamins (IU or mg/kg diet): DL-alpha tocopherol acetate, 100 mg; sodium menadione bisulphate, 25 mg; retinyl acetate, 20000 IU; DL-cholecalciferol, 2000 IU; thiamin, 30 mg; riboflavin, 30 mg; pyridoxine, 20 mg; cyanocobalamin, 0.1 mg; nicotinic acid, 200 mg; folic acid, 15 mg; ascorbic acid, 1000 mg; inositol, 500 mg; biotin, 3 mg; calcium panthotenate, 100 mg; choline chloride, 1000 mg, betaine, 500 mg. Minerals (g or mg/kg diet): cobalt carbonate, 0.65 mg; copper sulphate, 9 mg; ferric sulphate, 6 mg; potassium iodide, 0.5 mg; manganese oxide, 9.6 mg; sodium selenite, 0.01 mg; zinc sulphate, 7.5 mg; sodium chloride, 400 mg; calcium carbonate, 1.86 g; excipient wheat middlings.

^14^ Paramega PX, Kemin Europe NV, Belgium.

^15^ Monocalcium phosphate: 22% phosphorus, 16% calcium, Fosfitalia, Italy.

Diets were analysed for total amino acid content. Diet samples were hydrolysed in 6M HCl at 116°C for 2 h in nitrogen-flushed glass vials. Samples were then pre-column derivatised with Waters AccQ Fluor Reagent (6-aminoquinolyl-N-hydroxysuccinimidyl carbamate) using the AccQ Tag method (Waters, USA). Analyses were done by ultra-high-performance liquid chromatography (UPLC) in a Waters reversed-phase amino acid analysis system, using norvaline as an internal standard. During acid hydrolysis asparagine is converted to aspartate and glutamine to glutamate, so the reported values for these amino acids (Asx and Glx) represent the sum of the respective amine and acid. Tryptophan was not determined, since it is partially destroyed by acid hydrolysis. The resultant peaks were analysed with EMPOWER software (Waters, USA). The AA profile of the experimental diets is presented in Table 2.

**Table 2:** Amino acid composition (mg/g diet) of experimental diets. Trp was not analysed. Values are means ± SD.

	Experimental diets		
	CTRL	Arg1	Arg2
*IAA* ^1^			
Arg	41.6 ± 1.5	56.3 ± 0.7	64.8 ± 1.7
His	10.2 ± 0.4	10.5 ± 0.1	9.7 ± 0.1
Lys	31.2 ± 1.3	31.5 ± 1.5	32.8 ± 0.9
Thr	18.4 ± 0.3	18.9 ± 0.5	18.2 ± 0.1
Ile	21.3 ± 0.1	20.8 ± 0.1	20.9 ± 0.0
Leu	42.5 ± 0.0	41.2 ± 0.1	42.1 ± 0.1
Val	23.2 ± 0.1	22.9 ± 0.3	22.6 ± 0.1
Met	9.9 ± 0.3	10.0 ± 0.1	9.8 ± 0.1
Phe	23.4 ± 1.1	23.5 ± 0.1	22.1 ± 0.4
*DAA* ^2^			
Cys	2.8 ± 0.0	2.1 ± 0.1	2.4 ± 0.0
Tyr	21.1 ± 1.0	20.1 ± 0.2	19.4 ± 0.3
Asx	44.2 ± 2.1	45.2 ± 2.2	45.8 ± 1.3
Glx	103.3 ± 3.1	103.0 ± 5.0	101.4 ± 1.9
Ala	25.9 ± 0.7	26.0 ± 0.9	26.4 ± 0.3
Gly	34.8 ± 0.7	36.2 ± 1.6	34.0 ± 0.4
Pro	34.4 ± 0.2	33.4 ± 0.3	33.2 ± 0.1
Ser	24.5 ± 0.3	25.3 ± 1.0	24.0 ± 0.4
Tau	0.7 ± 0.0	0.6 ± 0.0	0.7 ± 0.0

^1^Indispensable amino acids;

^2^Dispensable AA amino acids

### 2.2 Fish

All trials were carried out at the indoor experimental facilities of the Instituto de Acuicultura Torre de la Sal (IATS-CSIC, Castellón, Spain). Non vaccinated fingerlings of 0.6 g initial body weight were purchased from a commercial hatchery (Grupo Tinamenor, Santander, Spain) and acclimatized for more than two months to IATS experimental facilities. Fish were fed over the course of this period (March-June 2014) with the CTRL diet in a flow-through system with aerated seawater under natural photoperiod (12 h light/ 12 h dark) and temperature (22.95°C ± 0.9) at IATS-CSIC latitude (40°5N; 0°10E). Water parameters were daily monitored with oxygen levels always higher than 85% saturation and unionized ammonia below toxic levels (< 0.05 mg l^-1^).

### 2.3 Feeding trial and sampling

One hundred and fifty fish of 12.17 ± 0.17 g average weight were randomly distributed into six 500 l tanks. Dietary treatments were randomly assigned to duplicate groups and fish were fed to visual satiety two times per day, 6 days per week) for 15 or 29 days. At the end of these two periods, 5 fish per tank (n = 10 per diet) were euthanized by overexposure to the anaesthetic MS-222 (Sigma, Saint Louis, USA). Specimens were weighed and blood was collected from the caudal vein with heparinized syringes. An aliquot of fresh blood was used for the respiratory burst assay and the remaining blood was centrifuged at 3000 × *g* for 20 min at 4°C to obtain plasma. Total visceral, mesenteric fat and liver weight were recorded. Subsequently, pieces of spleen, anterior (AI) and posterior (PI) intestine were immediately taken and frozen in liquid nitrogen. Plasma and tissue samples were kept at -80°C until further analyses. All procedures were approved by the Ethics and Animal Welfare Committee of Institute of Aquaculture Torre de la Sal and carried out in a registered installation (code 36271-42-A) in accordance with the principles published in the European animal directive (2010/63/EU) and Spanish laws (Royal Decree RD53/2013) for the protection of animals used in scientific experiments. In all lethal samplings, fish were decapitated after 3-aminobenzoic acid ethyl ester (MS-222, 100 μg ml^-1^) over-exposure, and all efforts were made to minimize suffering.

### 2.4 Bacterial inoculum preparation and challenge dose validation

*Vibrio anguillarum* serotype O1 (strain Lab 1), isolated from diseased European sea bass was cultured in tryptic soy agar (TSA, Pronadisa, Madrid, Spain) supplemented with NaCl at a final concentration of 1% (TSA-1) at 24°C for 24 hours.

Eight different doses were tested in a pre-challenge experiment in order to validate a suitable infective dose for the bacterial challenge. Juvenile European sea bass, held in 90 l tanks at an average temperature of 22.3°C, were intracoelomically (i.c.) injected with 0.1 ml of bacterial suspensions in phosphate-buffered saline (PBS, pH 7.4) ranging from 4.7 × 10^3^ to 1 × 10^6^ colony forming units (CFU) ml^-1^ (8–10 fish/dose) [16]. Negative control fish received 0.1 ml of PBS. Mortalities were monitored for 8 days and were considered due to *V*. *anguillarum* only if the inoculated bacterium was recovered in pure culture from internal organs. Kidney and liver samples collected from moribund fish were directly streaked onto TSA-1 plates. For identification of the pathogen, a slide agglutination test with the corresponding antiplasma was used. The dose producing mortalities between 40 and 50% (LD_40-50_) was chosen for the bacterial challenges.

### 2.5 Bacterial challenge

At the end of the first feeding period (15 days), 40 fish per dietary treatment were lightly anesthetized with clove oil (1:10,000), i.c challenged with the previously determined dose of *V*. *anguillarum* and distributed in triplicate 90 l tanks (n = 10). A group of 10 fish per dietary treatment was injected with PBS, as a control of the experimental handling. Fish were fed the same diets along the post-challenge period. The second bacterial challenge was performed 29 days after the beginning of the feeding trial, following the same procedure as in the first challenge. In both challenges, mortalities were monitored daily (every two hours) until no more mortalities were observed for a minimum of two consecutive days, so the trial was terminated 8 days post-challenge. Fish showing signs of disease (fish near the water surface, slowly swimming around the air stone or motionless at the bottom of the tank) were humanely sacrificed by overexposure to the anaesthetic as previously mentioned. Post-mortem examination was performed as described above.

### 2.6 Respiratory burst of circulating leucocytes

The respiratory burst was assessed in circulating leucocytes at the end of each feeding period following the method described by Nikoskelainen et al. [17]. Briefly, 4 μl of fresh blood were added to 96 μl of HBSS (Hanks’ Balanced Salt Solution, pH 7.4) in a white flat-bottomed 96-well plate and incubated with 100 μl of a freshly prepared luminol suspension (2 mM luminol in 0.2 M borate buffer pH 9.0, with 2 μg ml^-1^ PMA) for 1 h at 24–25°C. Each sample was run in duplicate and read against a blank into which no blood was added. Luminol-amplified chemiluminescence was measured every 3 min in a plate luminescence reader (TECAN) for generation of kinetic curves. Each sample was run in duplicate and the integral luminescence in relative light units (RLU) was calculated.

### 2.7 Innate humoral parameters

Plasma bactericidal activity was measured according to Graham et al. [18] with some modifications [19]. A suspension of *Photobacterium damselae* subsp. *piscicida* (20 μl, 1 × 10^6^ CFU ml^-1^) was added to 20 μl of plasma in duplicate wells of a U-shaped 96-well plate. HBSS was added instead of plasma to serve as positive control. After an incubation period of 2.5 h at 25°C, 25 μl of 3-(4,5 dimethyl-2-yl)-2,5-diphenyl tetrazolium bromide (1 mg ml^-1^, Sigma) were added to each well and plates were incubated for more 10 minutes at 25°C. Then, 200 μl of dimethyl sulfoxide (Sigma) were added after centrifugation at 2000 × *g* for 10 min. The absorbance of the formed, resuspended formazan was read at 560 nm in a Synergy HT (Biotek) microplate reader. Total bactericidal activity is expressed as the percentage of killed bacteria, calculated from the difference between the samples and the positive control (100% living bacteria).

Total plasma nitrite and nitrate content was measured using a Nitrate/Nitrite colorimetric kit (Roche Diagnostics GmbH, Mannheim, Germany) by adapting it to a 96-well plate and by following manufacturer’s instructions. Since both these compounds are derivatives of endogenously produced NO, they are indicative of NO amount in plasma. Briefly, 100 μl of plasma were added in duplicate to 50 μl of reduced nicotinamide adenine dinucleotide phosphate (NADPH) followed by the addition of 4 μl of nitrate reductase. A blank was produced by adding distilled water instead of plasma. Absorbance at 540 nm was read after 30 min incubation at 25°C. Afterwards, 50 μl of sulfanilamide and an equal volume of N-(1-naphthyl)-ethylenediamine dihydrochloride were added to each well. The mixture was allowed to stand at 25°C for 15 min and absorbance was read at 540 nm. Total nitrite levels were calculated from a previously prepared sodium nitrite standard curve.

### 2.8 Gene expression analysis

Spleen, AI and PI were taken from fish fed the experimental diets for 29 days. Total RNA was extracted using a MagMAX™-96 total RNA isolation kit (Life Technologies, Carlsbad, CA, USA). RNA yield was 50–100 μg with 260 and 280 nm UV absorbance ratios (A260/280) of 1.9–2.1. Reverse transcription (RT) of 500 ng total RNA was performed with random decamers using a High-Capacity cDNA Reverse Transcription Kit (Applied Biosystems, Foster City, CA, USA) according to manufacturer’s instructions. Negative control reactions were run without reverse transcriptase and real-time quantitative PCR was carried out on a CFX96 Connect™ Real-Time PCR Detection System (Bio-Rad, Hercules, CA, USA) using a 96-well PCR array layout designed for simultaneously profiling a panel of 30 genes under uniform cycling conditions. Genes were selected for their involvement in immune response and arginine metabolism (Table 3). Among the 30 genes, 24 genes were novel for European sea bass and their sequences, derived from the IATS-Nutrigroup transcriptomic database (www.nutrigroup-iats.org/seabassdb), were uploaded to GenBank (KM225766-KM225790, S1 Table). Controls of general PCR performance were included on each array, being performed all the pipetting operations by means of the EpMotion 5070 Liquid Handling Robot (Eppendorf, Hamburg, Germany). Briefly, RT reactions were diluted to convenient concentrations and the equivalent of 660 pg of total input RNA was used in a 25 μL volume for each PCR reaction. PCR-wells contained a 2× SYBR Green Master Mix (Bio-Rad) and specific primers at a final concentration of 0.9 μM were used to obtain amplicons of 50–150 bp in length (S2 Table).

**Table 3:** Immune-related genes analysed by real-time PCR.

Gene name	Symbol	GenBank Accession number
Argininosuccinate lyase	*ASL*	KM225766*
Argininosuccinate synthase	*ASS*	KM225767*
Arginase-2, mitochondrial	*ARG2*	KM225768*
Glycine amidinotransferase, mitochondrial	*GATM*	KM225769*
S-adenosylmethionine decarboxylase	*AMD1*	KM225770*
Ornithine decarboxylase	*ODC1*	KM225771*
Diamine acetyltransferase 1	*SAT1*	KM225772*
Spermine oxidase	*SMOX*	KM225773*
Nitric oxide-associated protein 1	*NOA1*	KM225774*
Nitric oxide-inducible gene protein	*NOXIN*	KM225775*
Nitric oxide synthase-interacting protein	*NOSIP*	KM225776*
Interleukin 1-β	*IL–1β*	AJ311925
Interleukin 8	*IL–8*	KM225777*
Interleukin 10	*IL–10*	DQ821114
Interleukin 20	*IL–20*	KM225779*
Interleukin 34	*IL–34*	KM225780*
Tumour necrosis factor-α	*TNF-α*	DQ070246
C-C chemokine receptor type 3	*CCR3*	KM225781*
C-C chemokine receptor type 9	*CCR9*	FN665390
Atypical chemokine receptor 4	*CCR11*	KM225782*
T-cell surface glycoprotein CD3 zeta chain	*CD247*	KM225783*
T-cell surface glycoprotein CD8 beta	*CD8b*	KM225784*
Myeloid differentiation primary response protein MyD88	*MyD88*	KM225785*
Myeloid cell surface antigen CD33	*CD33*	KM225786*
Macrophage colony-stimulating factor 1 receptor	*CSF1R*	KM225787*
Macrophage migration inhibitory factor	*MIF*	FN582353
Monocyte to macrophage differentiation factor	*MMD*	KM225788*
Interferon regulatory factor 8	*IRF8*	KM225789*
Nuclear factor NF-kappa-B p100 subunit	*NFKB2*	KM225790*
β-Actin	*ACTB*	AY148350

*New sequences for European sea bass are labelled with an asterisk.

The program used for PCR amplification included an initial denaturation step at 95°C for 3 min, followed by 40 cycles of denaturation for 15 s at 95°C and annealing/extension for 60 s at 60°C. The efficiency of PCR reactions was always higher than 90%, and negative controls without sample templates were routinely performed for each primer set. The specificity of reactions was verified by analysis of melting curves (ramping rates of 0.5°C/10 s over a temperature range of 55–95°C), and linearity of serial dilutions of RT reactions. Fluorescence data acquired during the PCR extension phase were normalized using the delta–delta Ct method (Livak and Schmittgen, 2001). Β-Actin was tested for gene expression stability using GeNorm software (M score = 0.21) and it was used as housekeeping gene in the normalization procedure. Fold-change calculations were done in reference to the expression ratio between Arg1 or Arg2 and CTRL fish (values >1 indicate up-regulated genes in Arg1 or Arg2 fish; values <1 indicate down-regulated genes in Arg1 or Arg2 fish). For comparing the mRNA gene expression level of a panel of genes in a given dietary treatment, all data values were in reference to the expression level of interleukin 10 in CTRL fish, which was arbitrarily assigned a value of 1.

### 2.9 Statistical Analysis

Results are expressed as means ± standard error of the mean (SEM). Data were analysed for normality and homogeneity of variances and, when necessary, transformed before being treated statistically. Analysis of fish performance, respiratory burst, plasma bactericidal capacity and NO content were performed by two-way analysis of variance (ANOVA) with dietary treatment and time of sampling as main variables, whereas gene expression data were analysed by one-way ANOVA with dietary treatment as the sole variable. Whenever significant differences were found, a multiple-comparisons Tukey HSD test was performed to identify significantly different groups. For every test, the level of significance chosen was P < 0.05. All data were analysed with STATISTICA (StatSoft, Inc. 2013, version 12) for WINDOWS.

## Results

### 3.1 Fish performance

Final fish weight was higher in fish reared for 29 days compared to those sampled at 15 days, regardless of the dietary treatment (Table 4). By contrast, the viscerosomatic index (VSI) was significantly lower in individuals fed Arg2 compared to fish fed Arg1 at day 15, whereas an increase in VSI was observed in animals fed Arg2 from day 15 to 29. Regarding the specific growth rate (SGR) and the feed conversion rate (FCR) no differences were observed among dietary treatments. Due to technical constraints, FCR data from 15 days are not presented.

**Table 4:** Data on the performance of European sea bass sampled 15 or 29 days after being fed three different diets. Values are means ± SEM (n = 10)

Parameters	15 days			29 days			P-value^1^		
	CTRL	Arg1	Arg2	CTRL	Arg1	Arg2	Time	Diet	Time × Diet
Final weight (g)	15.56 ± 0.49	18.21 ± 0.89	16.18 ± 0.66	21.55 ± 1.81	22.46 ± 1.74	23.16 ± 1.27	0.00	NS	NS
VSI^2^	11.81 ± 0.56^ab^	12.45 ± 0.43^a^	10.76 ± 0.30^b^*	11.88 ± 0.45	11.75 ± 0.29	12.51 ± 0.21	NS	NS	0.00
MSI^3^	5.47 ± 0.48	5.62 ± 0.34	4.68 ± 0.28	5.83 ± 0.63	4.66 ± 0.29	5.61 ± 0.37	NS	NS	NS
HSI^4^	1.95 ± 0.14	1.88 ± 0.12	1.60 ± 0.11	1.60 ± 0.10	1.90 ± 0.18	1.94 ± 0.25	NS	NS	NS
SGR^5^	1.69 ± 0.21	2.58 ± 0.34	1.80 ± 0.28	2.12 ± 0.32	2.25 ± 0.27	2.40 ± 0.20	NS	NS	NS
FCR^6^	-	-	-	0.87 ± 0.03	0.98 ± 0.04	0.92 ± 0.01	-	NS	-

^1^ P values were obtained from two-way analysis of variance. Different superscript letters in each row indicate significant differences between diets, within the same sampling time; asterisks denote differences between sampling time, within the same dietary treatment (Tukey HSD post-hoc test, P < 0.05)

^2^ Viscerosomatic index = (Viscera weight / final weight) × 100

^3^ Mesenteric fat-somatic index = (Mesenteric fat weight / final weight) × 100

^4^ Hepatosomatic index = (Liver weight / final weight) × 100

^5^ Specific growth rate = 100*((ln (Final weight) − ln (Initial weight)) / time)

^6^ Feed conversion ratio = Weight increase / (Feed intake × % dry matter)

### 3.2 Bacterial challenge

**Fig 1:**
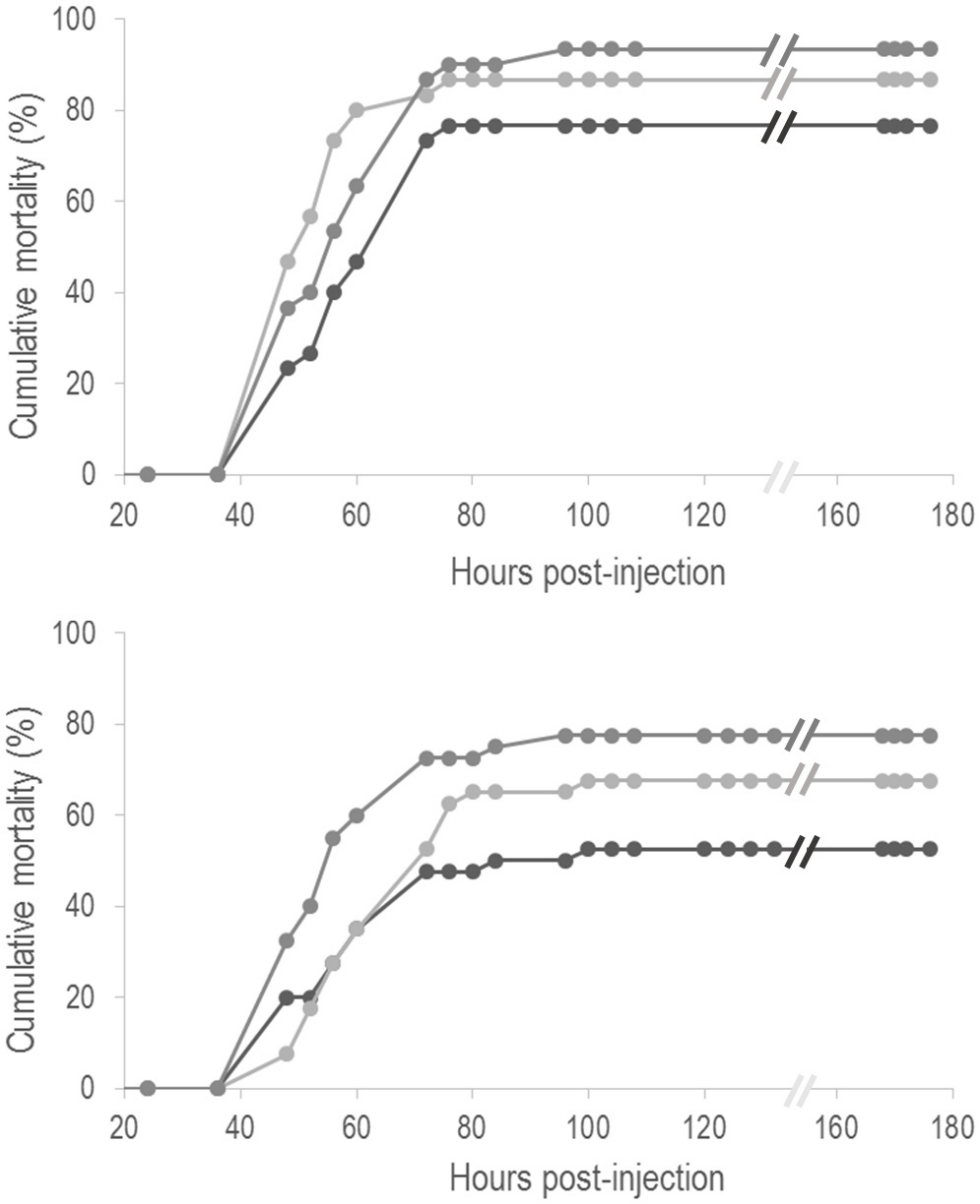
Cumulative mortality of European sea bass i.c. injected with *V*. *anguillarum* serotype O1 after 15 (1.5 × 10^4^ CFU ml^-1^, a) or 29 days (3 × 10^3^ CFU ml^-1^, b) of feeding with CTRL (black line), Arg1 (light grey line) or Arg2 (dark grey line) diets. Values are means of triplicate tanks (n = 30). SEM and sham injected fish are not presented for the clarity of the graphs.

Mortality started two days after bacterial challenge, regardless of the dietary treatment or the feeding time (Fig 1a and 1b). No mortality was observed in fish injected with PBS (data not shown). Concerning the first challenge (day 15 of feeding trial), the infective dose, which was based on the pre-challenge trial, was around 1.5 ×10^4^ CFU ml^-1^. The highest death numbers occurred in Arg1 and Arg2 dietary groups with 86.7% and 93.3% of mortality, respectively (Fig 1a). High mortalities, though lower, were recorded in the CTRL group (76.7%). As mortality was higher than expected in the CTRL group (probably due to an increase in mean water temperature—from initial temperature 22.3°C to final temperature 23.6°C), a lower dose was used for the second bacterial challenge (day 29 of feeding). Fish were injected with 3 × 10^3^ CFU ml^-1^ and mortality was lower than in the first challenge (Fig 1b). Fish fed Arg1 and Arg2 diets were more susceptible to the infection compared to those fed the CTRL diet, showing mortalities of 67.5%, 77.5% and 52.5%, respectively.

### 3.3 Respiratory burst of circulating leucocytes

**Fig 2:**
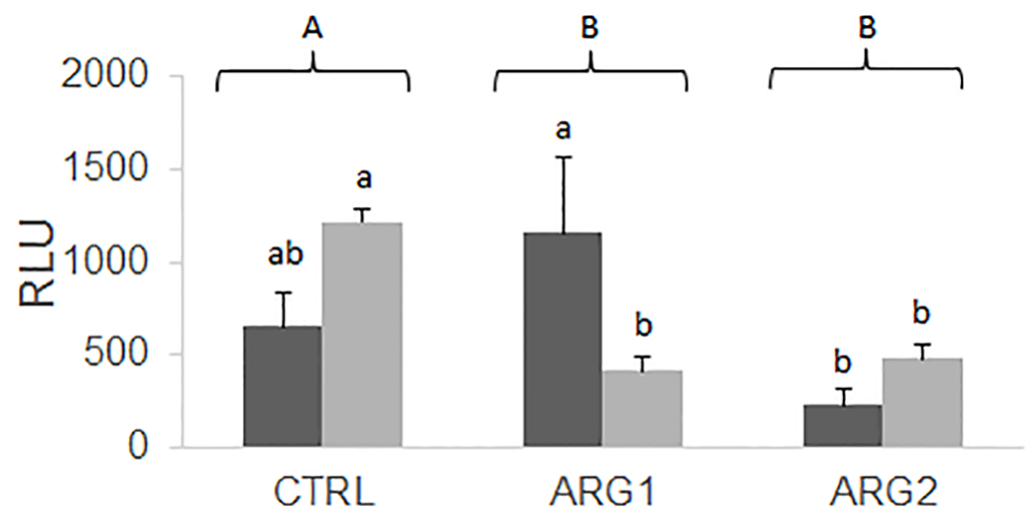
Respiratory burst activity of circulating leucocytes of European sea bass fed different diets for 15 (black columns) or 29 (grey columns) days. Values represent means ± SEM of relative light units (n = 6). Different capital letters stand for statistically significant differences between diets regardless of feeding time. Different low case letters stand for statistically significant differences between diets within the same feeding time (Two-way ANOVA; P < 0.05)

Dietary arginine significantly decreased the respiratory burst of circulating leucocytes, regardless of the supplementation level, when fish were fed for 29 days (Fig 2). Furthermore, the response of fish fed the Arg2 diet was also lower when fed only 15 days. An interaction between time and dietary treatment was also observed in fish fed Arg1 and Arg2 diets compared to individuals fed the CTRL diet at day 29.

### 3.4 Innate humoral parameters

**Fig 3:**
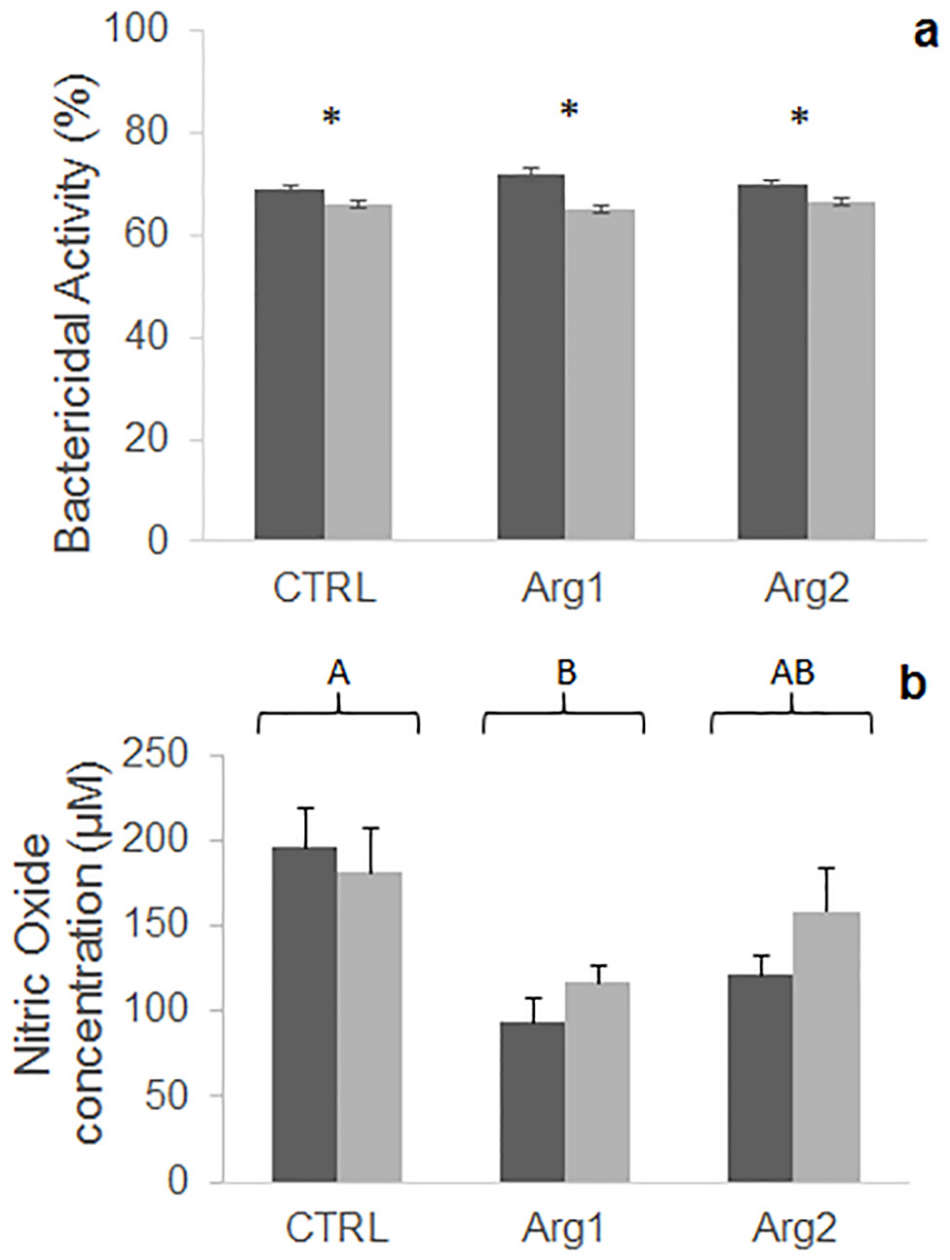
Bactericidal activity (a) and nitric oxide content (b) in plasma of European sea bass fed different diets for 15 (black columns) or 29 (grey columns) days. Values represent means ± SEM (n = 6). Asterisks denote differences attributed to feeding time within each diet. Different capital letters stand for statistically significant differences between diets regardless of feeding time (Two-way ANOVA; P < 0.05)

No dietary effects were observed on total plasma bactericidal activity (Fig 3a). However, a slight, but significant decrease was observed between the two feeding times in all diet groups. In contrast, decreased levels of NO were observed in Arg1 and Arg2 fish, though only significantly for the lowest supplementation level (Fig 3b). No effect of feeding time was observed on this parameter.

### 3.5 Gene expression

**Fig 4:**
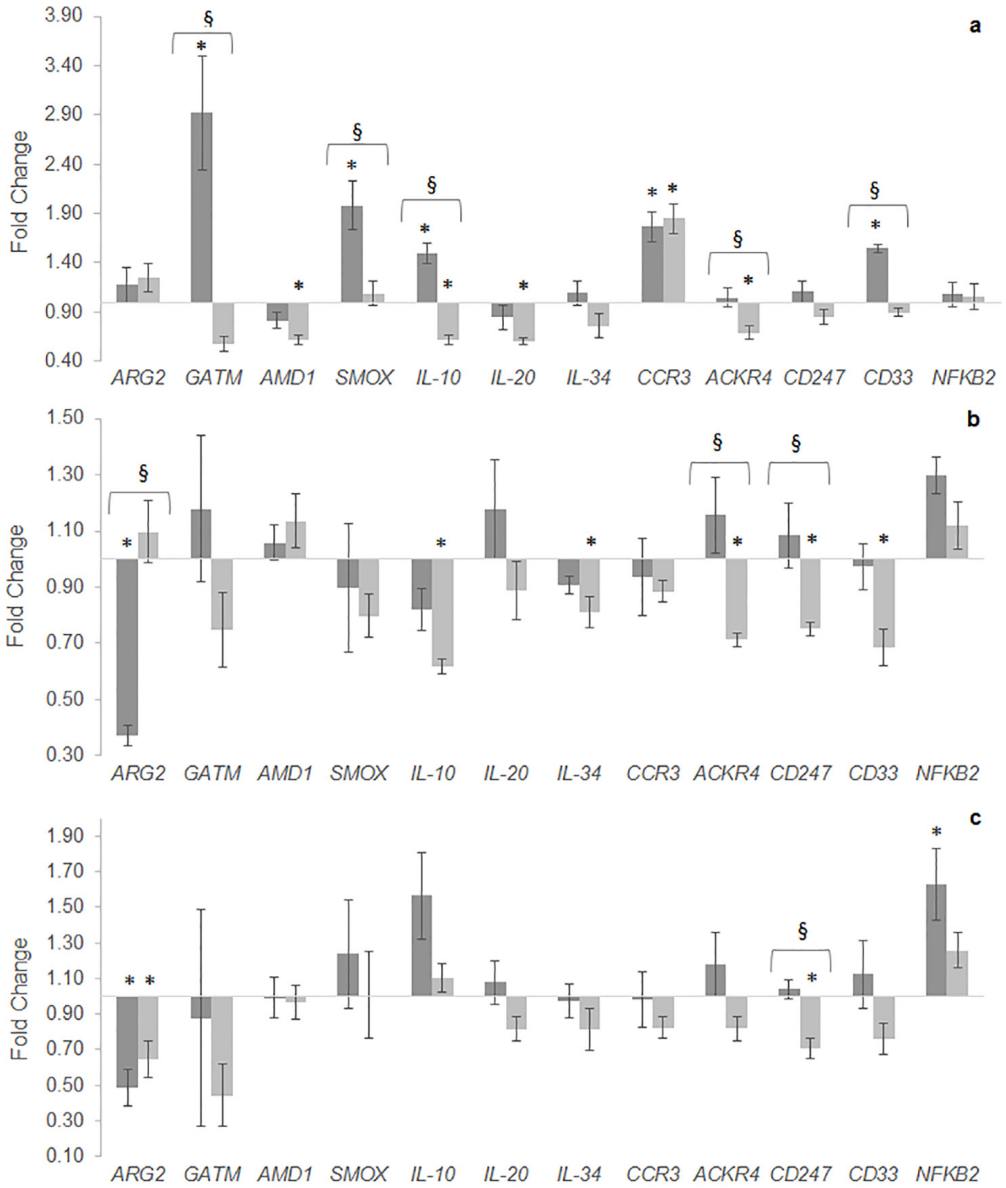
Quantitative expression of immune-related genes in spleen (a), anterior (b) and posterior (c) intestine of European sea bass fed different diets for 29 days. Data are presented as means ± SEM (n = 6). Bars represent fold change of Arg1 (dark grey columns) and Arg2 (light grey columns) relatively to the CTRL group, previously normalized to endogenous β-Actin expression levels. Asterisks indicate significant differences relatively to CTRL group; section sign indicates differences between arginine supplementation levels (One-way ANOVA; P < 0.05)

The relative expression of the 30 studied genes is shown in S1 File: Tables A, B and C (in spleen, AI and PI, respectively). To simplify data interpretation, only differently expressed genes are represented as fold change in Fig 4. Spleen retrieved the highest number of modulated genes, in comparison to both intestinal portions (Fig 4a). Arginase 2 (*ARG2*) expression was not affected in this tissue by any dietary treatment, but mRNA transcripts of the enzymes glycine amidinotransferase mitochondrial (*GATM*) and spermine oxidase (*SMOX*) were up-regulated in Arg1 fish compared to CTRL ones. Interleukin 10 (*IL-10*), membrane-bound molecules such as C-C chemokine receptor type 3 (*CCR3*) and myeloid cell surface antigen (*CD33*) transcripts were up-regulated in Arg1 fish, as well, whereas *CCR3* expression level also increased in Arg2 fish compared to CTRL fish. In contrast, S-adenosylmethionine decarboxylase (*AMD1*), *IL-10*, interleukin 20 (*IL-20*) and the atypical chemokine receptor 4 (*ACKR4*) transcripts decreased in Arg2 fish compared to CTRL diet. When comparing supplementation levels, Arg2 fish showed lower gene expression levels of *GATM*, *SMOX*, *IL-10*, *ACKR4* and *CD33* than Arg1 fish.

Dietary treatments did not affect expression of *GATM*, *AMD1*, *SMOX*, *IL-20* nor *CCR3* in the AI, whereas a decrease in *ARG2* transcripts was observed in Arg1 fish compared to CTRL fish (Fig 4b). Furthermore, mRNA expression levels of *IL-10*, *IL-34*, *ACKR4*, T-cell surface glycoprotein *CD3* zeta chain (*CD247*) and *CD33* were down-regulated in Arg2 fish. Regarding *ACKR4* and *CD247*, expression patterns were significantly lower than both CTRL and Arg1 fish.

Regarding gene expression patterns of the PI, a significant decrease of *ARG2* transcripts was denoted on both dietary groups relatively to the CTRL group, while *CD247* gene expression decreased in Arg2 fish (Fig 4c). By contrast, nuclear factor κB (*NFKB2*) mRNA levels augmented in Arg2 fish. Moreover, the expression levels of *CD247* were lower in Arg2 fish, compared to Arg1 specimens.

## Discussion

Immunomodulation mediated by arginine has received a special attention in the last years [20–22]. Arginine is an essential AA with versatile functions including protein synthesis, production of urea, synthesis of polyamines, proline and agmatine and it is also involved in endocrine and reproduction regulatory processes [23]. Therefore, its involvement in fish immune response may be essential for managing fish health throughout the full rearing cycle. Thus, it is important to understand the delicate balance of the immune response, in which a complex network of mechanisms is orchestrated by several external and internal factors. Arginine supplementation did not affect fish growth in this study. When juvenile gold pompano (*Trachinotus ovatus*) were fed six graded levels of arginine for eight weeks, fish growth was firstly increased and then depressed with the highest supplementation levels [24]. The same effect was observed by Zhou et al. [25] in juvenile yellow catfish (*Pelteobagrus fulvidraco*) fed diets with different levels of arginine for 84 days. The fact that such increase in growth was not observed in the present study may be related to the shorter feeding time (15 and 29 days).

Several studies have considered arginine administration as a strategy that improves the immune response, and, more specifically, the inflammatory process: in fish, previous studies have shown that several factors of the innate immune response, such as the extracellular superoxide anion production, lysozyme activity and neutrophil oxidative radical production were stimulated upon dietary arginine supplementation [26]. In turbot (*Scophthalmus maximus*), high rearing density caused immunosuppressive effects that were counteracted by dietary arginine supplementation [27]. Furthermore, Chen and co-authors [28] suggested that arginine contributed to an enhanced immune response of Jian carp and higher survival against *Aeromonas hydrophila* based on the up-regulation of pro-inflammatory gene expression. However, the results of the present study showed an opposite effect of dietary arginine supplementation in European sea bass. Indeed, bacterial challenges with *V*. *anguillarum* demonstrated a dose-dependent increased susceptibility of fish fed arginine supplemented diets. Thus, the highest mortalities occurred in the Arg2 groups and the lowest ones in CTRL fish. Mortality after bacterial challenge was also increased in golden pompano (*Trachinotus ovatus)* when dietary arginine supply was higher than that considered optimum [24]. This is perhaps indicative that arginine surplus might either improve or impair growth, immune response and disease resistance.

In the present study, measurements of gene expression and immune-related factors explained, to a certain extent, the observed higher disease susceptibility. One of the concurrent decreased immune factors in Arg1 and Arg2 fish was the respiratory burst of circulating leucocytes. The respiratory burst of phagocytes is part of the first line of cellular defence mechanisms and involves the production of reactive oxygen intermediates (ROI). This intracellular production of superoxide anion is a well-studied antimicrobial mechanism in fish that can be modulated by several factors [29] and therefore its decrease could be considered an immune-depression component. This is crucial in the current bacterial challenge, as V. *anguillarum* is able to inhibit leucocyte respiratory burst of European sea bass [30]. Thus, those animals with a reduced ability to produce ROI would be particularly jeopardized as they would display fewer obstacles for this bacterial invasion. The current results are in accordance with those obtained for other fish species, in which extracellular superoxide anion production of head kidney phagocytes was decreased such as in channel catfish (*Ictalurus punctatus*) fed 2–4% arginine supplemented diets, while no changes were detected in both phagocyte intracellular superoxide anion production and blood neutrophil respiratory burst [31]. By contrast, all respiratory burst values were decreased in fish fed an arginine deficient diet (0.5%). Authors attributed the lower levels of this free radical to a non-activated cells state. However, that would explain superoxide levels similar to the control group, but not lower values. Thus, the fact that, similar to the present work, superoxide anion production was lower indicates an inhibitory effect of arginine. Arginine does not play any direct role in the respiratory burst, and it has been demonstrated that an absence of arginine has no influence on granulocyte functions, including oxidative burst, in murine and human models [32]. However, it was demonstrated *in vitro* that induction of iNOS increases ROI production in murine macrophages [33]. Thus, the lower levels of plasmatic NO also observed in our study are in accordance with lower ROI production. Another explanation for the lower respiratory burst response could be the involvement of arginine metabolism in myeloid cells [11] which impairs both cell proliferation and activation. As a consequence, the production of superoxide anion would be compromised.

Another decreased immune factor was plasmatic NO, though only significantly in Arg1 fish. At first glance, this might appear contradictory. Since NO is synthesized from L-arginine by the catalytic action of NO synthases (NOS), it would be expected to find higher levels of this molecule when its precursor is more available. However, arginine is also substrate for arginase, which converts L-arginine into L-ornithine and can also be metabolized by the enzyme *gatm* into creatine. Therefore, supplementing the diet with L-arginine would decrease the competition between the three enzymes by providing extra substrate for them. The transcriptomic results of the genes involved in these metabolic pathways can clarify what occurred in the present study. First of all, a clear down-regulation of *ARG2* was detected in the PI of Arg1 and Arg2 fish, and also in the AI of Arg1 fish. *ARG2* is an extra-hepatic arginase involved in the production of ornithine, as precursor for polyamines, glutamate and proline, and in the regulation of arginine for NO synthesis [34]. Further, in various teleosts, liver arginase (also named ARG I) activity is induced following several weeks of fasting, as increased arginase activity may be important in AA catabolism in fasting fish [35, 36]. The observed *ARG2* down-regulation may have also been affected by a negative feedback set by the increased polyamine turnover. Indeed, *SMOX*, which is involved in the recycling of spermine (a polyamine) back to spermidine, was up-regulated in the spleen and PI of Arg1 fish, although only significantly in the spleen. Accordingly, *AMD1* was equally down-regulated in the spleen of these fish, which supports this hypothesis. The transcription of *GATM* was also up-regulated in the spleen and AI of Arg1 fish, even though not significantly in the latter tissue. Consequently, an increase in creatine could be expected. Several studies have suggested an anti-inflammatory role of creatinine: *in vitro* impairment of neutrophil adhesion to human endothelial cells upon creatine supplementation [37]; inhibitory effect on the expression of pathogen receptors in the membrane of human macrophages [38]. Creatine has also been classified as antioxidant [39]. Therefore, a putative increase of creatine biosynthesis would further support our findings regarding the lower plasma NO content, but further studies are needed to corroborate this hypothesis. Concerning the third group of enzymes, NOS, no differences were observed regarding *NOSIP* transcripts, probably due to the decreased NO production, as this protein promotes de translocation of eNOS (endothelial NOS, a constitutive NOS) from plasma membrane to intracellular sites. Similarly, *NOXIN* expression was not changed, which is not unexpected, as this gene is strongly induced by different NO donors in mammals. *NOXIN* plays an anti-apoptotic role, thus when the *NOXIN* gene is inactivated or down-regulated, cells exhibit higher levels of apoptosis than their counterparts with normal levels of *NOXIN* [40]. Currently, iNOS of European sea bass is unknown, therefore future studies should address if this inducible form of NOS [41] was indeed unchanged.

In mammalian models, under resting conditions, little arginine is used by myeloid cells and they do not express the major arginine metabolizing enzymes, iNOS and arginase. Thus, dietary arginine supplementation cannot enhance myeloid cell function in the absence of disease. It is only after stimulation that arginine transport into the myeloid cell is greatly increased. Some myeloid cells expressing arginase regulate T-cell function through arginine depletion. In addition, arginase is induced in myeloid cells by T-helper 2 (Th2) cytokines, such as IL-4 and IL-13, and also by IL-6, IL-10, TGF-β, prostaglandins and catecholamines [42]. Further studies are needed to understand all these complex interplaying regulatory factors in fish and how they are modulated after bacterial challenge to better understand the responses obtained before challenge.

The expression profile of immune-related genes differed in fish fed arginine supplemented diets and also supports the higher mortality observed in these groups. In teleosts, spleen is an important haematopoietic organ where melanomacrophages phagocytise and detain blood-borne antigens for a long period of time [43]. This tissue exhibited the highest number of differentially expressed genes, followed by AI and PI. The last portion of fish gut is characterized by a diffused presence of leucocytes in both the lamina propria and the epithelium [44], generally known as the gut-associated lymphoid tissue (GALT). The low modulation of gene expression in the PI may be related to an absence of a stimulatory event, for instance, a bacterial infection, which would certainly lead to immune cells activation. In the present study, pro- and anti-inflammatory interleukins (*IL-10*, *IL-20*, *IL-34*) were in general lower in Arg2 fish, revealing a broad inhibition of the inflammatory response, particularly in the spleen. Only *IL-10* was up-regulated in the spleen in Arg1 fish. Furthermore, along with other observed genes, its expression in the spleen of fish given the lowest supplementation was significantly higher than in Arg2 animals. The same pattern was observed in broiler chickens fed arginine-supplemented diets, in which both the expression of *IL-1β* (pro-inflammatory) and *IL-10* (anti-inflammatory) were down-regulated in a dose-dependent manner after a LPS challenge [45]. TGF-β expression, another important anti-inflammatory cytokine in fish, was up-regulated upon infection in Jian carp fed the highest level (24.5 g/kg of diet) of arginine supplementation, which translated in higher disease susceptibility after bacterial challenge [28]. Interleukins are mediators of both mounting and resolution of the inflammatory response [46]. Anti- and pro-inflammatory cytokines are essential for an efficient and correctly assembled inflammation. Anti-inflammatory cytokines are produced as a negative feedback to the secretion of other inflammatory mediators, thereby, avoiding an excessively harmful inflammatory response [47]. Furthermore, expression of arginase and iNOS is reciprocally influenced by Th1 and Th2 cytokines, and therefore activation of each of these cytokines may be restricted to separate subsets of myeloid-derived cells, termed alternative activation [42].

While changes in gene expression of cellular-produced compounds were more evident in the spleen, indicators of immune-related cells presence were more modulated in the AI of individuals fed the arginine diets. Indeed, mRNA levels of cell markers such as *ACKR4*, *CD247* and *CD33* dropped substantially in fish fed Arg2, suggesting that arginine was able to depress the cellular immune status of these fish, either by directly affecting cell proliferation or by impairing immune cells differentiation. The expression of *NFKB2*, a transcriptional factor directly related to the onset of the inflammatory response, was down-regulated in chickens treated with arginine supplementation [45]. By contrast, mRNA levels of *NFKB2* were up-regulated in the PI of Arg1 fish. *NFKB2* is normally present in the cell and can be rapidly activated either as result of a cascade of events following pathogen receptors activation [48], or by inflammatory mediators such as NO or pro-inflammatory cytokines. In the present context, where no stimulus was present, the up-regulation of this gene cannot be related to the enhancement of the immune response and remains to be clarified.

The inhibitory effect of arginine supplementation was also observed in Jian carp where both *in vivo* and *in vitro* arginine supplementations counteracted LPS-induced inflammatory responses [14]. Moreover, this experiment allowed the researchers to associate the inhibitory effects of arginine to a decrease in the expression of the LPS-recognizing receptor, TLR4, and two other molecules downstream of the activated cascade. The same authors suggested this might be a “protective effect” of arginine against LPS-induced inflammation, whereas other authors referred to arginine mode of action as an “alleviation” of the immune challenge caused by *Salmonella enterica* [49]. However, this issue should be addressed with care at least in fish, since the involvement of arginine in immunity is somehow contradictory. Pro-inflammatory signals and mediators are necessary for the establishment of an efficient immune reaction against a threat; so, their inhibition should not be seen as advantageous for the host. A balanced supplementation should improve the immune response while avoiding the damages of an excessive response.

## Conclusions

The present study clearly shows inhibition of some immune mechanisms in the European sea bass as result of dietary arginine supplementation, which led to higher disease susceptibility. These results may be due to the direct role of arginine on cell activation and differentiation leading to a restrained humoral response, or to the action of arginine on cell communications by inhibiting the production of pro-inflammatory mediators. This study provides additional information for deciphering the two-edged effects of a dietary surplus of arginine, as arginine supplementation may produce widely different biological responses depending on the disease process where it is provided. As in human studies [42], we are therefore still in the process of identifying in fish which processes are benefited by arginine-supplemented diets. In fish, the efficiency and balance of the immune system are highly dependent and susceptible to several external and internal varying factors. It is, therefore, of utmost importance to have full knowledge of each specific situation (e.g. developmental stage, fish species, etc.) and conditions (e.g. water temperature, densities, water quality, etc.), while supplementing aquafeeds aiming the improvement of fish health.

## Supporting Information

S1 Table Characteristics of new assembled sequences of European sea bass according to BLAST searches. (PDF)

S2 Table Forward and reverse primers for real-time PCR. (PDF)

S1 File Relative expression of genes involved in the immune response or arginine metabolism in the European sea bass spleen (A), anterior intestine (B) and posterior intestine (C) after 29 days of feeding trial. Different letters denote significant differences between dietary treatments (One-way ANOVA; P < 0.05)
(PDF)
