# Supplementary material for: European Sea Bass (Dicentrarchus labrax) Immune Status and Disease Resistance Are Impaired by Arginine Dietary Supplementation
Source: PLoS One. 2015 Oct 8;10(10):e0139967. doi: 10.1371/journal.pone.0139967 (PMC4598043; doi:10.1371/journal.pone.0139967)
Supplement: S1 Table — (PDF) [file pone.0139967.s001.pdf]

European sea bass (*Dicentrarchus labrax*) immune status and disease resistance are impaired by arginine dietary supplementation

Rita Azeredo[1,2*], Jaume Pérez-Sánchez[4], Ariadna Sitjà-Bobadilla[5], Belén Fouz[6], Lluis Tort[3], Cláudia Aragão[7], Aires Oliva-Teles[1,2], Benjamín Costas[1*]

[1]Centro Interdisciplinar de Investigação Marinha e Ambiental (CIIMAR), Universidade do Porto, Rua dos Bragas 289, 4050-123 Porto, Portugal.

[2]Departamento de Biologia, Faculdade de Ciências da Universidade do Porto (FCUP), 4169-007 Porto, Portugal.

[3]Departament de Biologia Cel·lular, Fisiologia Animal i Immunologia, Universitat Autònoma de Barcelona, Bellaterra, Spain.

[4]Nutrigenomics and Fish Growth Endocrinology Group, Institute of Aquaculture Torre de la Sal, IATS-CSIC, 12595 Ribera de Cabanes , Castellón, Spain.

[5]Fish Pathology Group, Institute of Aquaculture Torre de la Sal, IATS-CSIC, 12595 Ribera de Cabanes , Castellón, Spain.

[6]Department of Microbiology and Ecology, Faculty of Biology, University of Valencia, Dr Moliner 50, 46100 Burjassot, Valencia, Spain.

[7]Centro de Ciências do Mar, Universidade do Algarve, Campus de Gambelas, edf. 7, 8005-139 Faro, Portugal

***Corresponding authors:**

Rita Azeredo

Benjamín Costas

Email addresses: mleme@ciimar.up.pt; bcostas@ciimar.up.pt

S1 Table. Characteristics of new assembled sequences of European sea bass according to BLAST searches.

Contig	Size (nt)	Annotation[a]	Best match[b]	E[c]	CDS[d]	GenBank
L12_87226	2192	*ASL*	XP_004550300	0.0	154-1551	KM225766
L12_83771	1616	*ASS*	XP_003445281	0.0	<1-1242	KM225767
L1_28894	366	*ARG2*	BAF46063	7e-64	1->366	KM225768
L1_75798	1283	*GATM*	XP_004066965	5e-159	<1-689	KM225769
L12_83188	1663	*AMD1*	XP_007556077	0.0	284-1294	KM225770
L1_53546	811	*ODC1*	XP_004539948	5e-90	333->811	KM225771
L1_63507	918	*SAT1*	XP_003456292	3e-36	696->918	KM225772
L12_86945	2132	*SMOX*	XP_006787188	0.0	26-1741	KM225773
L12_75839	1285	*NOA1*	XP_004571208	0.0	<1-1115	KM225774
L1_44713	512	*NOXIN*	XP_006779966	3e-63	135->512	KM225775
L12_77157	1336	*NOSIP*	XP_005464991	3e-145	119-1033	KM225776
L2_36836	616	*IL-8*	AGR27883	2e-59	98-415	KM225777
L2_54923	496	*IL-20*	XM_004545365	6e-73	<1-456	KM225779
L2_69424	989	*IL-34*	BAM36385	6e-111	<1-512	KM225780
L12_90140	4399	*CCR3*	XP_005460244	4e-147	943-1971	KM225781
L12_82260	1585	*CCR11*	CBN82022	0.0	190-1293	KM225782
L12_77739	1356	*CD247*	XP_005457870	2e-29	290-685	KM225783
L12_73038	1123	*CD8b*	CBN81109	7e-133	116-754	KM225784
L12_83581	1697	*MyD88*	ADM25313	0.0	144-1016	KM225785
L3_76602	534	*CD33*	ACQ58565	8e-79	<1->534	KM225786
L12_87296	2199	*CSF1R*	CAJ18352	2e-164	<1-819	KM225787
L12_88792	2641	*MMD*	XP_005739998	3e-157	1418-2149	KM225788
L12_87913	2359	*IRF8*	AHB59740	0.0	127-1395	KM225789
L12_87846	2214	*NFKB2*	ABP35928	1e-75	<1-504	KM225790

[a]Gene identity determined through BLAST searches:

[b]Best BLAST-X protein sequence match (lowest E value): *ASL*, Argininosuccinate lyase; *ASS*, Argininosuccinate synthase; *ARG2*, Arginase-2 mitochondrial; *GATM*, Glycine amidinotransferase, mitochondrial; *AMD1*, S-adenosylmethionine decarboxylase; *ODC1*, Ornithine decarboxylase; *SAT1*, Diamine acetyltransferase 1; *SMOX*, Spermine oxidase; *NOA1*, Nitric oxide-associated protein 1; *NOXIN*, Nitric oxide-inducible gene protein; *NOSIP*, Nitric oxide synthase-interacting protein; *IL-8*, Interleukin 8; *IL-20*, Interleukin 20; *IL-34*, Interleukin 34; *CCR3*, C-C chemokine receptor type 3; *CCR11*, Atypical chemokine receptor 4; *CD247*, T-cell surface glycoprotein *CD3* zeta chain; *CD8b*, T-cell surface glycoprotein CD8 beta; *MyD88*, Myeloid differentiation primary response protein MyD88; *CD33*, Myeloid cell surface antigen CD33; *CSF1R*, Macrophage colony-stimulating factor 1 receptor; *MMD*, Monocyte to macrophage differentiation factor; *IRF8*, Interferon regulatory factor 8; *NFKB2*, Nuclear factor NF-kappa-B p100 subunit.

[c]Expectation value.

[d]Codifying sequence.
