# Supplementary material for: European Sea Bass (Dicentrarchus labrax) Immune Status and Disease Resistance Are Impaired by Arginine Dietary Supplementation
Source: PLoS One. 2015 Oct 8;10(10):e0139967. doi: 10.1371/journal.pone.0139967 (PMC4598043; doi:10.1371/journal.pone.0139967)
Supplement: S2 Table — (PDF) [file pone.0139967.s002.pdf]

European sea bass (*Dicentrarchus labrax*) immune status and disease resistance are impaired by arginine dietary supplementation

Rita Azeredo[1,2*], Jaume Pérez-Sánchez[4], Ariadna Sitjà-Bobadilla[5], Belén Fouz[6], Lluis Tort[3], Cláudia Aragão[7], Aires Oliva-Teles[1,2], Benjamín Costas[1*]

[1]Centro Interdisciplinar de Investigação Marinha e Ambiental (CIIMAR), Universidade do Porto, Rua dos Bragas 289, 4050-123 Porto, Portugal.

[2]Departamento de Biologia, Faculdade de Ciências da Universidade do Porto (FCUP), 4169-007 Porto, Portugal.

[3]Departament de Biologia Cel·lular, Fisiologia Animal i Immunologia, Universitat Autònoma de Barcelona, Bellaterra, Spain.

[4]Nutrigenomics and Fish Growth Endocrinology Group, Institute of Aquaculture Torre de la Sal, IATS-CSIC, 12595 Ribera de Cabanes , Castellón, Spain.

[5]Fish Pathology Group, Institute of Aquaculture Torre de la Sal, IATS-CSIC, 12595 Ribera de Cabanes , Castellón, Spain.

[6]Department of Microbiology and Ecology, Faculty of Biology, University of Valencia, Dr Moliner 50, 46100 Burjassot, Valencia, Spain.

[7]Centro de Ciências do Mar, Universidade do Algarve, Campus de Gambelas, edf. 7, 8005-139 Faro, Portugal

***Corresponding authors:**

Rita Azeredo

Benjamín Costas

Email addresses: mleme@ciimar.up.pt; bcostas@ciimar.up.pt

S2 Table. Forward and reverse primers for real-time PCR.

Gene name	Symbol	GenBank	*Eff*[1]	MT[2]	Product length[3]	Primer sequence
**Argininosuccinate lyase**	*ASL*	KM225766	94.3	79.2	54	F TGA GGC TGT GGC TGC GAG ATG C<br>R GCT GTA GGG AAT TGT TCG TCA GGG TTG AGA
**Argininosuccinate synthase**	*ASS*	KM225767	97.3	79.1	52	F GCC AAT GCT GTG TAT GAG GAC CGA TA<br>R GCC GTG CTA CCG CAG TTC C
**Arginase-2, mitocondrial**	*ARG2*	KM225768	88.5	81	79	F GAG CAC GGT CCT AAA GTC AT<br>R CAA AGT CGT GGA CAG AGT AGT
**Glycine amidinotransferase, mitochondrial**	*GATM*	KM225769	96.5	78.5	74	F GGA ATT GAG TGG ATG CGT CGT CAT<br>R GGG TTA GGG TCC TTG AAT GAG ATT ATG TG
**S-adenosylmethionine decarboxylase**	*AMD1*	KM225770	99.3	79.1	63	F CTG ACG GAA CTT ACT GGA CCA TC<br>R CGA AGC TGA CGT AGG AGA ACT C
**Ornithine decarboxylase**	*ODC1*	KM225771	99.9	78.8	69	F GGG CTG TAG TTA TGA CAC TGG CAT CC<br>R GCT GAA TCT CCA TCT TGC TTG CAC AGT
**Diamine acetyltransferase 1**	*SAT1*	KM225772	91.6	78.2	55	F GCA TCA TCG CTG AAA TCC AAG GAG AGA ACA<br>R CCA ACC ACC TTC AGG CCG TCA CT
**Spermine oxidase**	*SMOX*	KM225773	95.6	80	57	F CAC GGC TGC CAA CCT CTG AT<br>R CTC GTC CTC GCA CTC CAC ATA AAT G
**Nitric oxide-associated protein 1**	*NOA1*	KM225774	92.9	81.1	62	F CCA TCC ACA AAG CCA CCA TAT CG<br>R GGG AAA CTT CAG CAG GTT CAG AG
**Nitric oxide-inducible gene protein**	*NOXIN*	KM225775	94.1	79.1	72	F AGA GGT TGG TGG AGA ACT TGG ATG GA<br>R CGA CAG CCT TCA TCA ACA ATG TGG ATC T

S2 Table. Continued.

Gene name	Symbol	GenBank	*Eff*[1]	MT[2]	Product length[3]	Primer sequence
**Nitric oxide synthase-interacting protein**	*NOSIP*	KM225776	97.1	78.9	66	F GTC CAA GCC CAT CAA CCC ATT CAC<br>R CTG TCT GTC CTG CTC TTC TCA CCT C
**Interleukin 1-β**	*IL-1β*	AJ311925	91.4	79.2	73	F CAT GAG CGA GAT GTG GAG ATC CAA GAT<br>R CAT TGT CAG TGG GTG GTG GGT AAT C
**Interleukin 8**	*IL-8*	KM225777	92.4	79	70	F CAA TCA GCA GGG ACT ACA ACA CAC A<br>R CTG TCT GGA GGG ATG ATC CTT GAC T
**Interleukin 10**	*IL-10*	DQ821114	92.4	78.8	67	F CAG TGC TGT CGT TTT GTG GAG GGT TTC<br>R TCT CTG TGA AGT CTG CTC TGA GTT GCC TTA
**Interleukin 20**	*IL-20*	KM225779	99.6	79	60	F GCT AGA AAT AAA GGA GGC GGC ACA GAA GG<br>R CAG TCC AGC ACA GTG TCC AGT TCT C
**Interleukin 34**	*IL-34*	KM225780	97.5	79.3	58	F AGA ACC CGA CAG AGT GCC AGA GT<br>R CAG GAG GGA TTT TGG GGA CGC ATA TC
**Tumour necrosis factor-α**	*TNF-α*	DQ070246	95.5	80.2	57	F TCT ACA GCC AGG CGT CGT TCA G<br>R CCG CAC TTT CCT CTT CAC CAT CGT
**C-C chemokine receptor type 3**	*CCR3*	KM225781	94.1	81.1	88	F TGA CCT TCG ACC GAC ACC TA<br>R ACA ATA CAG GAG ACT ACC GCA TAG C
**C-C chemokine receptor type 9**	*CCR9*	FN665390	94.7	78.4	69	F CCT GTG TGT CTG GCT TGT TTC TAC TCT C<br>R TCG CTC TTC ACC TGG GCA AAG ATA AAC TC
**Atypical chemokine receptor 4**	*CCR11*	KM225782	94.8	81	67	F TAC TTC TCT TCA CCC TGC CTT TCT G<br>R GCT GCC GAA CCC AAC TTC CA

S2 Table. Continued.

Gene name	Symbol	GenBank	*Eff*[1]	MT[2]	Product length[3]	Primer sequence
**T-cell surface glycoprotein CD3 zeta chain**	*CD247*	KM225783	99.8	78.6	81	F CTG ATG CGT CTG AAG AGA ATG GAG GC<br>R GTT CAA GCA CCT GGT AAG GAT CAG CAT C
**T-cell surface glycoprotein CD8 beta**	*CD8b*	KM225784	93.9	79.4	77	F AGT GAT CCC GCC AAC ATT ACC TCC TA<br>R TCT TCT TAG GGC AGC GAC AGA CT
**Myeloid differentiation primary response protein MyD88**	*MyD88*	KM225785	89.9	79.2	71	F CCA ATT CAG GTT GAT GAG GTT GAC A<br>R TCC TCC AGG GTG ATA CCA ATC C
**Myeloid cell surface antigen CD33**	*CD33*	KM225786	97.2	80.3	70	F CTG TTC ATT CAC CCA TCC TAG AG<br>R GGT CGA ACG ATG CCA GAT T
**Macrophage colony-stimulating factor 1 receptor**	*CSF1R*	KM225787	92.1	80	74	F CGG GCA GGA ACA GCT AAT CTA CCA<br>R ACT TGG GCT CAT CAC ACA CTT CAC
**Macrophage migration inhibitory factor**	*MIF*	FN582353	89.3	78.8	76	F GCT CCC TCC ACA GTA TTG GCA AGA T<br>R TTG AGC AGT CCA CAC AGG AGT TTA GAG T
**Monocyte to macrophage differentiation factor**	*MMD*	KM225788	88.9	77.4	59	F GGT CAT CTA CTT CTT CAT CGC TGC CTC CTA<br>R CCA ACT CTC GCA GGT TCA ACC AAG GT
**Interferon regulatory factor 8**	*IRF8*	KM225789	96.4	79.9	65	F TCT GAA GGC TGC CGA ATC TCC<br>R CTG TCT GAA CTG TAT AGG GCA CCA C
**Nuclear factor NF-kappa-B p100 subunit**	*NFKB2*	KM225790	92.5	78.8	55	F CTG GAG GAA ACT GGC GGA GAA GC<br>R CAG GTA CAG GTG AGT CAG CGT CAT C
**ß-Actin**	*ACTB*	AY148350	96.4	76.9	51	F TCC TGC GGA ATC CAC GAG A<br>R AAC GTC GCA CTT CAT GAT GCT

[1] Efficiency of PCR reactions were calculated from serial dilutions of tissue RT reactions in the validation procedure.
[2] Melting temperature
[3] Amplicon (nt)
