# Supplementary material for: European Sea Bass (Dicentrarchus labrax) Immune Status and Disease Resistance Are Impaired by Arginine Dietary Supplementation
Source: PLoS One. 2015 Oct 8;10(10):e0139967. doi: 10.1371/journal.pone.0139967 (PMC4598043; doi:10.1371/journal.pone.0139967)
Supplement: S1 File — Different letters denote significant differences between dietary treatments (One-way ANOVA; P < 0.05) (PDF) [file pone.0139967.s003.pdf]

European sea bass (*Dicentrarchus labrax*) immune status and disease resistance are impaired by arginine dietary supplementation

Rita Azeredo[1,2*], Jaume Pérez-Sánchez[4], Ariadna Sitjà-Bobadilla[5], Belén Fouz[6], Lluis Tort[3], Cláudia Aragão[7], Aires Oliva-Teles[1,2], Benjamín Costas[1*]

[1]Centro Interdisciplinar de Investigação Marinha e Ambiental (CIIMAR), Universidade do Porto, Rua dos Bragas 289, 4050-123 Porto, Portugal.

[2]Departamento de Biologia, Faculdade de Ciências da Universidade do Porto (FCUP), 4169-007 Porto, Portugal.

[3]Departament de Biologia Cel·lular, Fisiologia Animal i Immunologia, Universitat Autònoma de Barcelona, Bellaterra, Spain.

[4]Nutrigenomics and Fish Growth Endocrinology Group, Institute of Aquaculture Torre de la Sal, IATS-CSIC, 12595 Ribera de Cabanes , Castellón, Spain.

[5]Fish Pathology Group, Institute of Aquaculture Torre de la Sal, IATS-CSIC, 12595 Ribera de Cabanes , Castellón, Spain.

[6]Department of Microbiology and Ecology, Faculty of Biology, University of Valencia, Dr Moliner 50, 46100 Burjassot, Valencia, Spain.

[7]Centro de Ciências do Mar, Universidade do Algarve, Campus de Gambelas, edf. 7, 8005-139 Faro, Portugal

***Corresponding authors:**

Rita Azeredo

Benjamín Costas

Email addresses: mleme@ciimar.up.pt; bcostas@ciimar.up.pt

Table A. Relative expression of genes involved in the immune response or arginine metabolism in the European sea bass spleen after 29 days of feeding trial. Different letters denote significant differences between dietary treatments (One-way ANOVA; P < 0.05)

Genes	Diets			One-way ANOVA
	CTRL	Arg1	Arg2	
ASL	0.85 ± 0.056	1.10 ± 0.17	0.83 ± 0.077	0.21
ASS	0.15 ± 0.02	0.18 ± 0.03	0.14 ± 0.02	0.33
ARG2	0.19 ± 0.03	0.22 ± 0.03	0.24 ± 0.03	0.53
GATM	0.18 ± 0.03 [a]	0.70 ± 0.19 [b]	0.11 ± 0.01 [a]	0.00
AMD1	7.23 ± 0.84 [a]	5.92 ± 0.61 [ab]	4.5 ± 0.37 [b]	0.01
ODC1	0.5 ± 0.08	0.57 ± 0.08	0.49 ± 0.06	0.66
SAT1	2.62 ± 0.33	2.8 ± 0.37	2.65 ± 0.46	0.95
SMOX	0.3 ± 0.043 [a]	0.6 ± 0.07 [b]	0.33 ± 0.04 [a]	0.00
NOA1	1.04 ± 0.04	1.29 ± 0.13	1.05 ± 0.12	0.21
NOXIN	0.25 ± 0.03	0.32 ± 0.07	0.26 ± 0.03	0.63
NOSIP	5.34 ± 0.44	5.15 ± 0.46	4.54 ± 0.5	0.46
IL1β	0.04 ± 0.01	0.08 ± 0.01	0.06 ± 0.01	0.11
IL-8	0.17 ± 0.06	0.3 ± 0.05	0.14 ± 0.03	0.23
IL-10	1.02 ± 0.08 [a]	1.52 ± 0.11 [b]	0.63 ± 0.06 [c]	0.00
IL-20	0.63 ± 0.06 [a]	0.6 ± 0.09 [a]	0.38 ± 0.02 [b]	0.03
IL-34	11.76 ± 1.85	12.88 ± 1.45	8.93 ± 1.47	0.25
TNF-α	0.80 ± 0.11	0.72 ± 0.13	0.54 ± 0.04	0.23
CCR3	0.89 ± 0.10 [a]	1.56 ± 1.37 [b]	1.64 ± 0.13 [b]	0.00
CCR9	1.29 ± 0.19	1.50 ± 0.26	1.25 ± 0.22	0.71
ACKR4	3.49 ± 0.26 [a]	3.64 ± 0.34 [a]	2.40 ± 0.25 [b]	0.01
CD247	3.27 ± 0.32	3.61 ± 0.36	2.78 ± 0.24	0.18
CD8b	0.4 ± 0.05	0.38 ± 0.03	0.33 ± 0.04	0.40
MyD88	2.07 ± 0.15	2.53 ± 0.15	2 ± 0.19	0.08
CD33	45.17 ± 5.34 [a]	69.77 ± 2.04 [b]	40.92 ± 2.94 [a]	0.00
CSF1R	6.91 ± 1.1	6.68 ± 0.64	5.71 ± 0.67	0.54
MIF	2.5 ± 0.18	2.94 ± 0.29	2.81 ± 0.42	0.65
MMD	0.65 ± 0.04	0.67 ± 0.06	0.63 ± 0.05	0.84
IRF8	3.9 ± 0.45	4.21 ± 0.53	3.82 ± 0.43	0.84
NFKB2	10.46 ± 0.53	11.27 ± 1.25	11.01 ± 1.33	0.88

Table B. Relative expression of genes involved in the immune response or arginine metabolism in the European sea bass anterior intestine after 29 days of feeding trial. Different letters denote significant differences between dietary treatments (One-way ANOVA; P < 0.05)

**Genes**	**Diets**			**One-way ANOVA**
	CTRL	Arg1	Arg2	
ASL	2.26 ± 0.27	2.48 ± 0.33	1.81 ± 0.24	0.25
ASS	0.48 ± 0.05	0.54 ± 0.02	0.51 ± 0.07	0.81
ARG2	153.10 ± 16.55 [a]	58.02 ± 5.71 [b]	168.01 ± 16.81 [a]	0.00
GATM	446.84 ± 99.70	526.85 ± 117.12	334.53 ± 59.24	0.33
AMD1	15.84 ± 1.83	16.76 ± 0.97	17.99 ± 1.51	0.59
ODC1	2.48 ± 0.46	2.72 ± 0.77	2.18 ± 0.21	0.75
SAT1	7.43 ± 0.79	9.54 ± 1.00	7.84 ± 0.59	0.19
SMOX	9.48 ± 2.46	8.52 ± 2.16	7.56 ± 0.74	0.74
NOA1	6.66 ± 0.55	6.31 ± 0.66	6.35 ± 0.36	0.88
NOXIN	0.85 ± 0.07	0.71 ± 0.06	0.82 ± 0.05	0.25
NOSIP	27.49 ± 2.69	26.90 ± 3.44	24.65 ± 1.07	0.72
IL-1β	0.25 ± 0.10	0.24 ± 0.03	0.21 ± 0.03	0.86
IL-8	0.61 ± 0.12	0.67 ± 0.22	0.76 ± 0.11	0.82
IL-10	1.03 ± 0.13 [a]	0.85 ± 0.08 [ab]	0.64 ± 0.03 [b]	0.01
IL-20	0.84 ± 0.09	0.98 ± 0.15	0.74 ± 0.09	0.3
IL-34	2.97 ± 0.12 [a]	2.69 ± 0.10 [ab]	2.41 ± 0.17 [b]	0.04
TNF-α	2.68 ± 0.27	2.76 ± 0.26	2.24 ± 0.11	0.21
CCR3	7.93 ± 1.17	7.44 ± 1.08	7.02 ± 0.30	0.77
CCR9	7.80 ± 0.64	7.57 ± 0.89	7.07 ± 0.89	0.83
ACKR4	43.22 ± 3.18 [ab]	50.03 ± 5.84 [a]	30.84 ± 1.06 [b]	0.01
CD247	14.31 ± 0.46 [a]	16.86 ± 1.90 [a]	10.74 ± 0.32 [b]	0.00
CD8b	1.33 ± 0.08	1.02 ± 0.09	1.07 ± 0.12	0.10
MyD88	16.89 ± 3.09	15.18 ± 1.03	14.24 ± 0.66	0.59
CD33	7.34 ± 0.80 [a]	7.15 ± 0.60 [a]	5.03 ± 0.47 [b]	0.03
CSF1R	6.50 ± 1.11	6.09 ± 0.61	5.42 ± 0.38	0.57
MIF	57.34 ± 6.70	52.65 ± 5.30	59.67 ± 4.69	0.69
MMD	49 ± 5.51	52.47 ± 4.63	46.10 ± 3.62	0.62
IRF8	11.60 ± 1.98	11.48 ± 1.30	10.16 ± 0.60	0.7
NFKB2	24.98 ± 3.07	32.36 ± 1.63	27. 98 ± 2.11	0.12

Table C. Relative expression of genes involved in the immune response or arginine metabolism in the European sea bass posterior intestine after 29 days of feeding trial. Different letters denote significant differences between dietary treatments (One-way ANOVA; P < 0.05)

Genes	Diets			One-way ANOVA
	CTRL	Arg1	Arg2	
ASL	6.13 ± 0.79	6.40 ± 0.66	7.44 ± 1.79	0.76
ASS	1.52 ± 0.16	1.65 ± 0.21	1.46 ± 0.18	0.76
ARG2	88.79 ± 4.26 [a]	36.75 ± 9.06 [b]	46.74 ± 5.18 [b]	0.00
GATM	10.97 ± 4.80	76.46 ± 32.35	41.82 ± 21.13	0.26
AMD1	36.00 ± 2.41	35.76 ± 4.16	34.73 ± 3.47	0.96
ODC1	2.51 ± 0.49	2.48 ± 0.29	2.58 ± 0.23	0.98
SAT1	14.80 ± 2.39	17. 57 ± 2.33	14.65 ± 0.95	0.51
SMOX	22.33 ± 3.67	27.62 ± 6.82	22.48 ± 5.49	0.75
NOA1	9.06 ± 0.99	9.53 ± 1.23	9.36 ± 0.79	0.95
NOXIN	1.67 ± 0.24	1.50 ± 0.19	1.54 ± 0.10	0.78
NOSIP	41.45 ± 5.84	43.93 ± 5.46	38.90 ± 2.90	0.76
IL-1β	0.33 ± 0.09	0.33 ± 0.04	0.37 ± 0.05	0.85
IL-8	1.32 ± 0.28	1.17 ± 0.41	1.46 ± 0.42	0.87
IL-10	1.11 ± 0.22	1.73 ± 0.27	1.22 ± 0.09	0.09
IL-20	1.57 ± 0.30	1.70 ± 0.19	1.29 ± 0.11	0.43
IL-34	7.41 ± 0.97	7.21 ± 0.70	6.02 ± 0.89	0.48
TNF-α	4.09 ± 0.82	4.24 ± 0.60	4.02 ± 0.42	0.97
CCR3	15.38 ± 1.97	15.07 ± 2.38	12.67 ± 0.95	0.51
CCR9	25.58 ± 3.30	37.18 ± 6.25	22.59 ± 3.10	0.06
ACKR4	62.32 ± 7.72	73.59 ± 11.05	51.16 ± 4.28	0.13
CD247	29.12 ± 2.76 [a]	30.27 ± 1.56 [a]	20.53 ± 1.68 [b]	0.00
CD8b	2.61 ± 0.48	2.65 ± 0.69	1.89 ± 0.20	0.38
MyD88	26.13 ± 3.32	29.08 ± 2.67	24.75 ± 1.46	0.52
CD33	16.74 ± 1.83	18.84 ± 3.19	12.74 ± 1.49	0.19
CSF1R	11.43 ± 1.70	8.99 ± 0.80	7.67 ± 0.91	0.11
MIF	104.87 ± 9.33	94.40 ± 8.28	84.95 ± 4.47	0.22
MMD	62.09 ± 11.07	50.56 ± 12.24	51.71 ± 10.24	0.75
IRF8	13.24 ± 1.72	17.94 ± 2.77	15.85 ± 1.22	0.28
NFKB2	43.96 ± 7.18 [a]	71.62 ± 8.83 [a]	55.25 ± 4.31 [ab]	0.04
